# Smartphone Apps and Wearables for Health Parameters in Young Adulthood: Cross-Sectional Study

**DOI:** 10.2196/64629

**Published:** 2025-09-03

**Authors:** Gaia Leuzzi, Mirko Job, Aldo Scafoglieri, Marco Testa

**Affiliations:** 1 Department of Neurosciences, Rehabilitation, Ophthalmology, Genetics, Maternal and Child Health, University of Genoa, Campus of Savona, Via Magliotto, 2, Savona, 17100, Italy; 2 Department of Physical Education and Rehabilitation, Experimental Anatomy Research Group (EXAN), Vrije Universiteit Brussel, Brussels, Belgium

**Keywords:** exercise, mobile apps, diet, mental health, mHealth, apps, digital health, smartphones, wearables, parameters, young adulthood, adolescents, teenagers, young adult, cross-sectional study, public, involvement, interventions, active aging, strategies, healthy lifestyle, technology, physical activity

## Abstract

**Background:**

Fostering innovative and more effective interventions to support active aging strategies from youth is crucial to help this population adopt healthier lifestyles using technologies they are already familiar with. Mobile health (mHealth), particularly apps and wearables, represents a promising approach due to its versatility, ease of use, and ability to monitor multiple health variables simultaneously. Moreover, these devices offer opportunities for personalization and support in health behavior change, making them valuable tools for shaping healthy habits from a young age.

**Objective:**

This study aims to (1) investigate whether young adults (18‐26 years old) use apps or wearables to monitor or improve their health variables (ie, physical activity, diet, and mental health); (2) examine how they use them; (3) identify the most commonly used apps and wearables and the most frequently monitored health variables across these domains; and (4) evaluate the importance of different characteristics and functions of apps and wearables for health purposes.

**Methods:**

This cross-sectional study used a public involvement framework to enhance the research quality and was conducted through an anonymous web survey disseminated across Italy over a 3-month period. The survey consisted of 5 sections: (1) demographics, (2) mobile apps and wearable devices for physical activity and sports, (3) mobile apps and wearable devices for diet, (4) mobile apps and wearable devices for mental health, and (5) preferences regarding mobile apps and wearable devices. Participants were eligible if they were young adults who reported using at least one app or wearable device to monitor at least one health variable (eg, steps, training, sleep, calorie intake). No additional eligibility criteria were applied.

**Results:**

A total of 693 questionnaires were analyzed for aims 1 and 4, with the sample showing an equal gender distribution (females: 363/693, 52.4%). For aims 2 and 3, a total of 317 questionnaires were included. Participants using an app or wearable for physical activity accounted for 320 (46.2%), while 60 (8.7%) and 156 (22.5%) reported use for diet and mental health, respectively. Moreover, the frequency of use was predominantly on a daily basis, particularly for wearables. The app and wearable characteristics identified as most important were user-friendliness, free access to content, loading speed, and icon clarity.

**Conclusions:**

Findings suggest that Italian young adults, particularly women, predominantly use wearables over apps to track health data, with both being checked on a daily basis. Physical activity is the most frequently monitored domain, likely due to its ease of tracking, while diet and mental health receive less attention. Overall, these tools are used more for monitoring than for actively improving health-related variables. The most valued characteristics identified by young adults include ease of use, free access to all content, and fast loading speed. These insights should guide the design and refinement of digital health interventions targeting this population.

## Introduction

Promoting healthy aging, or “active aging” [1], helps prevent excessive pressure on medical systems in the forthcoming years [2], while improving both individual and collective health [3]. This strategy combines theoretical and practical interventions to teach people, starting from youth, how to monitor and enhance health through a virtuous and sustainable lifestyle [4]. Reaching a large population in real time is crucial for the success of this slow and consistent process. In the last decades, mobile health (mHealth) [5,6] interventions have provided general and personalized information to support decision-making and behavior change. In this context, mobile apps designed for smartphones are particularly popular among young people and adults [7], offering continuous data collection via built-in sensors and adaptability to different scenarios [8,9]. Given the rapid evolution of this technology, it is crucial to understand its use and effectiveness in creating sustainable and useful mHealth apps. To promote lasting behavioral changes for active aging, apps must specifically target young adults [10] and adapt to their needs. Educating young adults (18-26 years old) [11] about healthy lifestyles using familiar tools [12] encourages long-term adoption. Therefore, it is important to explore how apps and wearables for health purposes are used by this specific population to manage domains such as physical activity (PA), diet, and mental health. In particular, considering that health is defined as a state of complete physical, mental, and social well-being and not merely the absence of disease or infirmity [13], these domains might play a role in health promotion for early active aging strategies.

The Health Belief Model (HBM) is a useful framework for understanding health-related behaviors [14]. It suggests that individuals are more likely to engage in health-promoting actions, such as using apps or wearables, if they perceive susceptibility to health issues, recognize their severity, and believe the benefits outweigh the barriers. Cues to action, such as app notifications, and self-efficacy in performing healthy actions further drive behavior change. By incorporating these elements, apps and wearables can motivate young adults to adopt healthy habits, supporting early active aging strategies.

In 2023, Italy had 5.3 million young adults, with about 1 million overweight or obese, 2.3 million not practicing any sports, and 2.4 million with chronic diseases [15]. Hence, it is fundamental not only to monitor but also to help this population effectively manage and improve its health. Moreover, in 2023, students enrolled in secondary high schools in Italy were reported to be 2.7 million, while 1.8 million were enrolled in universities [15].

Additionally, identifying the most and least appreciated features of these apps and wearables, along with their functions, is crucial for creating preventive and educational interventions [16] that assist young people in monitoring and improving their health [17-19], well-being, and quality of life through familiar technology tailored to their needs and expectations.

Therefore, this study aims to (1) determine whether young adults use mobile apps and wearable devices to monitor and improve their health variables (ie, PA, diet, and mental health); (2) understand how young adults use mobile apps and wearable devices to monitor and improve these health variables; (3) assess the most used mobile apps and wearables in terms of brands, as well as the most monitored variables via both apps and wearables; (4) evaluate the most important functions and characteristics of mobile apps and wearable for health variables based on young adults’ opinion.

## Methods

### Study Design

This work consists of a cross-sectional study performed via an anonymous web survey distributed across the Italian territory. The survey was created in accordance with the International Handbook of Survey Methodology [20] and the Declaration of Helsinki [21], and was distributed via Microsoft Forms (Office 365 Suite), which ensures secure and ease of data collection, as well as anonymity in compliance with General Data Protection Regulation [22] policies. Before accessing the survey, each participant was presented with an informative note about the study and data processing.

Participation was completely voluntary, and completion of the survey could be interrupted at any time and for any reason, without the need for explanation; in such cases, no data were saved. The reporting of this manuscript follows the STROBE (Strengthening the Reporting of Observational Studies in Epidemiology) guidelines [23]. At each phase of the research, a habitual mobile app user was involved to ensure the “public involvement,” as discussed below.

### Ethical Considerations

Ethical approval for this study was granted by the University of Genoa’s Ethical Committee for Research (CERA2023.24, approved on April 27, 2023). The study adhered to good clinical practice guidelines for the ethical conduct of research involving human participants. Participants were not compensated for their involvement, as clearly stated in the information sheet and informed consent form provided before their participation. All participants reviewed the study details and provided informed consent before taking part. Data were anonymized at the point of collection, as the use of Microsoft Fforms allowed participants to complete the survey without submitting personal information unless explicitly requested by the researchers. Data were collected and stored in accordance with current regulations, in a password-protected digital archive accessible only to the researchers directly involved in the study.

### Web Survey

The web survey was created by a panel of 5 professionals from different fields, such as sports science and health, psychology, engineering, and physiotherapy, and a habitual mobile app user pursuing a master’s degree in communication sciences. Existing questionnaires were reviewed to identify relevant questions, but none were deemed suitable for this specific research. Consequently, a preliminary draft of the survey was developed for evaluation and subsequent validation. The final web survey was divided into 5 sections: (1) demographics; (2) mobile apps and wearable devices for PA and sport; (3) mobile apps and wearable devices for food and diet; (4) mobile apps and wearable devices for mental health; and (5) preferences regarding mobile apps and wearable devices. Once imported into Microsoft Forms, the questions were organized with branching logic, allowing participants to skip sections on unused mobile apps or wearable devices, thereby enabling faster and more efficient completion. Following the demographics section, each subsequent section began with an initial yes-or-no question, giving participants the option to either skip that section or proceed. The estimated total completion time was about 5 minutes. The full survey is available in Multimedia Appendix 1.

The web survey was assessed for face and content validity [24] by 10 potential participants who used mobile apps to monitor their PA, diet, or mental health. These individuals met the study’s inclusion criteria and were asked to provide feedback on the survey before its adoption in the data collection process, to ensure data reliability and quality. Specifically, each participant had either a face-to-face meeting or a video call with the same professional, who explained the research aims, provided the informative note and informed consent documents, and, once consent was obtained, initiated the survey completion. Afterward, participants were asked questions about the survey’s characteristics, and all notes and suggestions were recorded. The feedback received was subsequently discussed by the panel of experts to produce the final version of the web survey. Face and content validity assessments are crucial in survey creation to ensure that the main topic under investigation is correctly addressed and that the questions and answers are understandable to the target population. This ensures that the results can be accurately analyzed and compared with the research question. In particular, content validity is provided by the panel of experts in the field investigation, whereas face validity is assessed by potential participants. Additionally, to evaluate the internal consistency of the survey, a Cronbach α [25] analysis was performed. The results confirmed good internal consistency (Cronbach α=0.891), as values of 0.70 and above are generally considered acceptable. The scale ranges from 0 to 1, with 1 indicating the highest level of internal consistency. The higher the value obtained for the survey items, the better the internal consistency is considered to be. No other validation methods were applied to this survey.

### Public Involvement

Our work employed the “study-focused” public-involvement framework [26], considering the point of view of the target population (ie, young adults aged 18‐26 years using at least one app or wearable to monitor or improve PA, diet, or mental health) across all phases of the study [20]. The public representative was (1) a 23-year-old female student; (2) enrolled in the first year of a master’s degree program in communication science; and (3) actively engaged with a mobile app for PA and possessing previous experience in using a mobile app for diet. Given the exploratory nature of this study, which aimed to investigate the prevalence of app and wearable use for health purposes among young adults as a whole, and not among specific subgroups (eg, by gender), a single representative, male or female, of the target population was deemed sufficient and was not expected to influence the results. The focus was on understanding general behaviors rather than subgroup-specific patterns, such as those based on gender.

The public representative volunteered to actively participate in formulating the research question and played a crucial role in creating, validating, and distributing the web survey. After the data collection period, she was also involved in the data analysis and the overall interpretation of the results, which was fundamental for better understanding the needs of the target population and maximizing the value of the collected data. Moreover, the public representative contributed to the writing and review of the final draft of this paper.

### Participants

To answer the first and last aims (1 and 4) of the study, participants were considered eligible if they were young adults (ie, aged 18‐26 years) and could read and understand the informative note and the web survey.

For aims 2 and 3, participants were eligible if they used at least one mobile app or wearable device for PA, diet, or mental health, and could read and understand the informative note and the web survey.

No other limitations were set for the population or for the type of app or wearable device used.

### Setting and Modalities of Contact

The web survey was distributed via Microsoft Forms (Office 365 Suite) in July and from September to October 2023. Potential participants were reached through various modalities, including face-to-face, digital, and printed invitations (see Multimedia Appendix 2). Printed advertisements were placed in locations frequently visited by young adults, both education-related and not (eg, university campuses, local groups headquarters, libraries). Social media platforms (eg, Facebook, Instagram, WhatsApp), email, and high school and university mailing lists were also used to disseminate invitations to complete the survey. To maximize nationwide reach, all 73 universities in Italy were contacted. Of these, only 7 agreed to assist in distributing the survey. The list of universities was publicly available online, and each university’s website was visited to obtain a contact email to explain the study rationale and request assistance with its promotion. The universities and high schools that agreed to collaborate were free to choose the dissemination method they considered most appropriate. To help balance the potential sample of participants, voluntary and social groups were also invited to share the study invitations. Concerning email, researchers used addresses that were publicly available in the “Contacts” section of relevant websites to reach potentially interested groups. Additionally, participants were encouraged to share the invitation with others who might be interested in participating. The recruitment process aimed to reach a diverse and broad audience, including individuals both engaged and not engaged in education, and from different regions of the country. Efforts were made to maintain a balance in region of origin, gender representation, and age distribution within the predefined age range. As the web survey was anonymous, and to prevent multiple responses, the option to submit more than 1 questionnaire was disabled directly in the platform settings. To further ensure data reliability, the dataset was checked for duplicates in Microsoft Excel after data collection. In cases of potential duplicates, variables such as age, gender, height, weight, and education were examined; no duplicates were found.

### Statistical Analysis

Sociodemographic data were analyzed using descriptive statistics, with percentages reported where applicable, and continuous variables presented as mean (SD). Binomial logistic regression analyses were performed to assess the influence of gender and education level on the use of apps and wearables. The last section of the questionnaire was analyzed using different methods. The first method defined consensus as being reached for each statement when 2 conditions were met [27]: (1) at least 51% of participants responded with “quite important” or “very important,” and (2) the median score exceeded 2.5. In our case, considering the IQRs, if the median was above 2.5, it could be assumed that at least 75% of the responses fell in the neutral or positive categories (ie, 2, 3, and 4). The second method involved the calculus of a ratio value between positive and negative answers collected. Moreover, a descriptive evaluation of results obtained from the Likert-type scale was adopted.

For evaluating the main variables monitored through mobile apps and wearables, the variables were divided into specific areas of interest (ie, PA, diet, and mental health) and aggregated into categories based on their similarities. PA-related variables were divided into “PA metrics” (ie, daily steps, distance covered, daily activity, training, number of weekly trainings, calorie consumption, elevation, and speed/step per kilometer) and “physiological parameters” (ie, heart rate, weight, oxygen saturation, sleep, stress, rest, body composition, menstrual cycle monitoring, and respiratory frequency). Diet parameters were grouped into “nutritional metrics” (ie, calorie consumption, macronutrient count, and water) and “body metrics” (ie, weight and body composition). Finally, mental health parameters were categorized as “sleep,” “mental well-being” (ie, stress, mood, and emotions management), and “mindfulness and meditation practices” (ie, breathing, meditation, mindfulness). Additionally, the motivation behind the use of these mobile apps and wearables was presented, with results split between the 2 types of mHealth.

The last section of the questionnaire investigated mobile app and wearable preferences, and the results of each question were graphically reported. Participants were asked to indicate their level of agreement with each statement on a 5-point Likert-type scale. The possible answers were as follows: (0) “not important at all,” (1) “unimportant,” (2) “neutral,” (3) “quite important,” and (4) “very important”. The results were then aggregated into 3 levels of agreement: negative (ie, 0 and 1), neutral (ie, 2), and positive (ie, 3 and 4). Moreover, an analysis of IQRs was performed, and medians and the distribution of responses were reported.

### Bias

Young adults with different levels of education were contacted to reduce any possible selection bias. To better describe the target population, invitations were disseminated across the entire Italian territory without restrictions, with the aim of reaching participants with diverse characteristics such as age, gender, occupation, and education level. Other universities and several secondary high schools were also contacted to increase the number of responses. Detailed modalities of dissemination are reported in the “Setting and Modalities of Contact,” and study limitations are discussed in the “Strengths and Limitations” section.

## Results

A total of 821 responses were received, and after applying the inclusion and exclusion criteria, a final sample of 693 questionnaires was analyzed. Specifically, 42 questionnaires were excluded because participants did not consent to data treatment, 83 did not meet the study’s age range (ie, 18‐26), and 3 were excluded due to unusable data. Demographic data for the final sample are presented in Table 1.

**Table 1.** Demographic characteristics of the sample (n=693).

Characteristics	Values
Age (years), mean (SD)	21.2 (2.5)
Gender, n (%)	
Female	363 (52.4)
Male	329 (47.5)
Nonbinary	1 (0.1)
BMI (kg/m^2^), mean (SD)	22.3 (3.03)
Last educational degree, n (%)	
Secondary school diploma	95 (13.7)
High school diploma	414 (59.7)
Higher education	184 (26.6)
Not using any mobile app or wearable, n (%)	317 (45.7)
Physical activity (n=144), n (%)	
1 app	106 (73.6)
2 or more apps	38 (26.4)
Diet (n=52), n (%)	
1 app	45 (86.5)
2 or more apps	7 (13.5)
Mental health (n=45), n (%)	
1 app	37 (82.2)
2 or more apps	8 (17.8)

Data regarding the use of mobile apps or wearables for PA, diet, or mental health variables are reported in Table 2 and include only those participants who reported using at least one app or wearable to monitor at least one health variable (n=376).

**Table 2.** Total number and percentage of participants using mobile apps or wearables for physical activity, diet, and mental health (n=693)..

Variables	Yes	No	Eliminated^a^
Physical activity, n (%)	320 (46.2)	338 (48.8)	35 (5.1)
App and wearable, n	56 (8.1)	—^b^	—
Only app, n	88 (12.7)	—	—
Only wearable, n	176 (25.4)	—	—
Diet, n (%)	60 (8.7)	622 (89.8)	11 (1.6)
App and wearable, n	15 (2.2)	—	—
Only app, n	37 (5.3)	—	—
Only wearable, n	8 (1.2)	—	—
Mental health, n (%)	156 (22.5)	524 (75.6)	13 (1.9)
App and wearable, n	19 (2.7)	—	—
Only app, n	26 (3.8)	—	—
Only wearable, n	111 (16.0)	—	—

a Data from each section were eliminated if participants provided conflicting answers within that section.

b Not available.

Table 3 ranks and details the most used mobile apps and wearables by brand and further divides them into specific spheres of interest (ie, PA, diet, and mental health).

**Table 3.** Ranking by brand of the most used mobile apps and wearables (n=376).

App	Physical activity (n=320)		Diet (n=60)		Mental health (n=156)	
	App (n=144)	Wearable (n=232)	App (n=52)	Wearable (n=23)	App (n=45)	Wearable (n=130)
1, n (%)	Apple Health: 62 (43.0)	Apple Watch: 81 (35)	Yazio: 15 (28.8)	Apple: 8 (34.8)	Apple Health: 7 (15.5)	Apple: 39 (30)
2, n (%)	Samsung Health: 25 (17.4)	Xiaomi: 37 (16)	MyFitnessPal: 11 (21.1) FatSecret: 11 (21.1)	Xiaomi: 7 (30.4)	Samsung Health: 6 (13.3) Google Fit: 6 (13.3) · FitBit: 6 (13.3)	Amazfit: 35 (27)
3, n (%)	WeWard: 18 (12.5)	Amazfit: 25 (10.7) Garmin: 25 (10.7)	Samsung Health: 7 (13.5)	Samsung: 4 (17.6)	Calm: 5 (11.1)	Samsung: 12 (9.2) Garmin: 12 (9.2)
4, n (%)	Google Fit: 14 (9.7)	Samsung: 20 (8.6)	Apple Health: 5 (9.61)	Fitbit: 1 (4.3) Garmin: 1 (4.3) Huawei: 1 (4.3) Redmi: 1 (4.3)	Garmin Connect: 4 (8.9) Zepp Life: 4 (8.9) Headspace: 4 (8.9)	Huawei: 10 (7.6)
5, n (%)	Garmin Connect: 11 (7.6)	Fitbit: 12 (5.2) Huawei: 12 (5.2)	Macros: 3 (5.8) · Melarossa: 3 (5.8)	N/A^a^	Flo: 3 (6.7) Serenity: 3 (6.7)	Fitbit: 9 (6.8)
6, n (%)	Zepp Life: 9 (6.2)	Honor: 5 (2.2)	Lifesum: 2 (3.8)	N/A	N/A	Honor: 5 (3.8)
7, n (%)	Komoot: 7 (4.9)	Polar: 4 (1.7)	Google Fit: 1 (1.9) · Fitdays: 1 (1.9) Lose it: 1 (1.9)	N/A	Daylio: 1 (2.2) · Stoic: 1 (2.2) · Mindshift: 1 (2.2)	Google: 1 (0.8) Polar: 1 (0.8) Oura: 1 (0.8) · Real me: 1 (0.8) Liujo: 1 (0.8) Suunto: 1 (0.8) Whoop: 1 (0.8) Xiaomi: 1 (0.8)
Others, n (%)	A1^b^: 52 (36.1)	A2^c^: 11 (4.7)	N/A	N/A	N/A	N/A

a N/A: not applicable.

b A1: SweatCoin, Strava, Fitbit, iSkiTracker, Pedometer, MyWellness, My workout plan, Huawei health, Hevy, Relive, Pokemon Go, Fitdays, Adidas Running, Nike training, GloryFit, Nike Run, etc.

c A2: Oppo, Google, Real me, Redmi, Suunto, and Liujjo.

Furthermore, the frequency of use of both mobile apps and wearables was evaluated and is presented in Table 4.

**Table 4.** Frequency of use of mobile apps and wearables.

Variables	Physical activity		Diet		Mental health	
	Mobile app	Wearable	Mobile app	Wearable	Mobile app	Wearable
Total users, n	144	232	52	23	45	130
Every day, n (%)	53 (36.8)	148 (63.8)	31 (59.6)	16 (69.6)	16 (35.6)	91 (70.0)
5 or 6 times a week, n (%)	20 (13.9)	24 (10.3)	10 (19.2)	3 (13.0)	4 (8.9)	12 (9.2)
3 or 4 times a week, n (%)	31 (21.5)	27 (11.6)	2 (3.8)	2 (8.7)	8 (17.8)	10 (7.7)
1 or 2 times a week, n (%)	20 (13.9)	22 (9.5)	4 (7.7)	1 (4.3)	9 (20.0)	8 (6.2)
Less than once a week, n (%)	20 (13.9)	11 (4.7)	5 (9.6)	1 (4.3)	8 (17.8)	9 (6.9)

Moreover, motivation for the use of apps and wearables, as well as the health variables monitored via these devices, is reported in Multimedia Appendix 3. The vast majority of the sample did not report using premium or paid content (310/320, 96.9%, participants monitoring PA; 58/60, 96.7%, monitoring diet; 152/156, 97.4%, monitoring mental health). Those who did use these additional functionalities primarily sought more detailed information on specific aspects such as sleep, rest periods, training, macro- and micronutrients, or personalized advice based on their data. Similarly, community functions were not used by most participants (289/320, 90.3%, monitoring PA; 54/60, 90%, monitoring diet; 142/156, 91%, monitoring mental health; see Multimedia Appendix 4)

Additionally, an analysis of the frequency distribution of answers based on gender and level of education was conducted. Binomial logistic regressions were also performed on these parameters to explore possible associations between the use of apps and wearables and gender or education level. Regarding gender, the percentage of females monitoring PA is higher than that of males (216/693, 31.2% vs 138/693, 19.9%), while nonusers are 191 out of 693 (27.6%) for males and 147 out of 693 (21.2%) for females. A slight difference was also found in diet monitoring, with 325 out of 693 (46.9%) females and 297 out of 693 (42.9%) males reporting no monitoring. For mental health, gender differences were observed, with 105 out of 693 (15.2%) females and 64 out of 693 (9.2%) males reporting monitoring. Logistic regressions examining gender influence on the monitoring of PA, diet, and mental health revealed a slight positive correlation for males in each sphere. Specifically, the models account for 2.4% for PA, 1.0% for diet, and 1.1% for mental health. Full results of these binomial logistic regressions are reported in Multimedia Appendix 5. Regarding education level, the frequency distribution for females and males differed across categories: (1) secondary school diploma (females: 23/693, 3.3%; males: 72/693, 10.4%), (2) high school diploma (females: 228/693, 32.9%; males: 185/693, 26.7%), (3) bachelor (females: 100/693, 14.4%; males: 4/693, 0.6%), and (4) master’s degree (females: 12/693, 1.7%; males: 4/693, 0.6%). Logistic regression models explained 5.0% of the variance for PA, 0.5% for diet, and 2.4% for mental health. For PA, having a bachelor’s or master’s degree showed a slight negative influence on the use of apps or wearables, and the same pattern was observed for mental health, while for diet, only a master’s degree has a slight negative influence on use. Full results of these analyses are reported in Multimedia Appendix 5. Finally, none of the differences related to gender or education level were statistically significant (see Multimedia Appendix 5).

**Figure 1.**
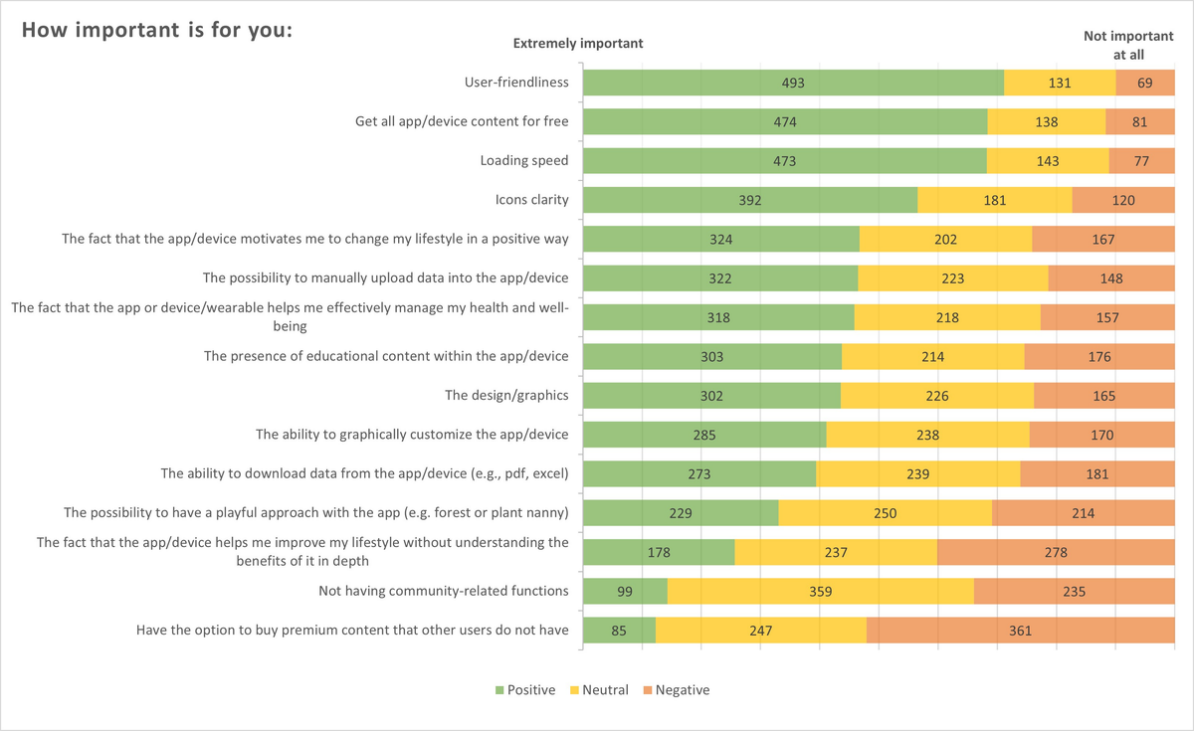
Mobile apps and wearables’ functions preferences (n=693).

For the fifth and final part of the questionnaire, all 693 participants were included in the analysis. Considering the assessment of the most important characteristics of mobile apps and wearables, 4 elements emerged as particularly important for young adults: (1) user-friendliness, (2) free access to all content, (3) loading speed, and (4) icon clarity. Figure 1 presents the aggregated responses for each question according to the level of attributed importance.

**Figure 2.**
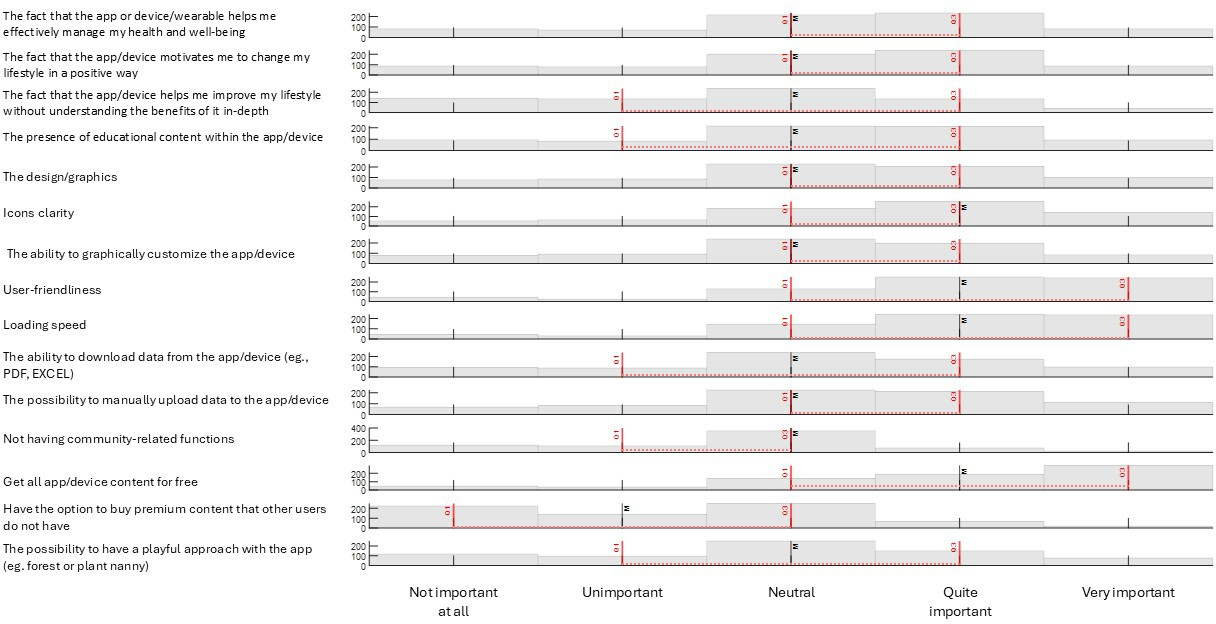
IQRs, medians, and distribution of responses for mobile apps and wearables’ functions preferences.

Figure 2 shows the distribution of responses, including IQRs and medians. Results are displayed for each of the original 5-point Likert scale options available to participants.

A second method of analysis was applied to the last section of the questionnaire, particularly to the data regarding elements considered important. The analysis evaluated the ratio of positive to negative answers, both with and without including neutral responses, as reported in Table 5.

**Table 5.** Ratio values of responses for mobile apps and wearables’ functions preferences.

Device’s characteristics	Positives/negatives	$\frac{positives+neutrals}{negatives+neutrals}$
User-friendliness	7145	3120
Get all app/device content for free	5852	2800
Loading speed	6143	2795
Icons clarity	3267	1904
The fact that the app/device motivates me to change my lifestyle in a positive way	1940	1469
The possibility of manually uploading data into the app/device	2176	1429
The fact that the app or device/wearable helps me effectively manage my health and well-being	2025	1425
The presence of educational content within the app/device	1722	1350
The design/graphics	1830	1326
The ability to graphically customize the app/device	1676	1282
The ability to download data from the app/device (eg, PDF, Excel)	1508	1219
The possibility to have a playful approach with the app (eg, forest or plant nanny)	1070	1032
The fact that the app/device helps me improve my lifestyle without understanding the benefits of it in depth	0.640	0.806
Not having community-related functions	0.421	0.771
Have the option to buy premium content that other users do not have	0.235	0.546

From this analysis, it can be observed that user-friendliness, loading speed, free access to all content, and icon clarity were confirmed as the most important elements for our sample. Moreover, the possibility of manually uploading data and the efficiency of the app or device in helping individuals manage their health were also indicated as potentially important. When neutral responses are also considered, the elements with the highest ratio values remain user-friendliness, free access to all content, loading speed, and icon clarity. Other ratio values were lower and may not warrant further investigation.

## Discussion

### Principal Findings

This study comprehensively investigated the use of apps and wearables to track and improve health variables among young adults. The average BMI of the sample fell within the normal range, consistent with national trends. In 2023, the Italian National Institute of Statistics reported that the majority of young adults, approximately 2.8 million individuals, had a BMI within the normal range [15]. While nearly half of the participants reported using these tools to track PA, only a minority used them to monitor diet or mental health. Conversely, almost half of the sample reported not using any app or wearable for health purposes. This might be due to a low perceived relevance of health monitoring, negative aspects associated with using such tools (eg, distress or fixation), or a lack of understanding or trust in these devices and the results they provide [28,29]. Another main issue in using apps and wearables for health is the manual input required for certain data, which leads to lower adherence and reduced usage of these tools [30-32]. The findings regarding diet are consistent with those reported by Hahn et al [33] in a study conducted on a comparable population. Similarly, the reported percentage of use for mental health apps aligned with previous literature [34,35], except for Borghouts et al [35], who reported a higher usage rate. Viewed through the lens of the HBM, PA may be the most frequently monitored health domain due to the greater perceived benefits of tracking it and the immediate, tangible feedback provided by step count, distance covered, or calories burned. In line with this, the perceived severity and susceptibility of problems related to sedentary behavior (eg, weight gain) may also serve as motivating factors. By contrast, monitoring diet and mental health presents greater barriers, as it often requires manual data entry [30], thereby reducing engagement, self-efficacy, and both the frequency and quality of data collection [31,32]. In particular, diet variables cannot be automatically monitored, especially from wearables. For mental health, automated tracking of variables such as sleep or stress through heart rate variability [36] provides an exception, as it requires less user interaction, fostering higher self-efficacy by simplifying behavior monitoring. To increase diet and mental health monitoring, digital assistants (eg, vocal assistants), artificial intelligence, and machine learning could be implemented into apps and wearables to automate tasks that are now manually required [37], lowering the need for users’ interaction and broadening the adoption also to other populations with lower digital skills.

The higher prevalence of app and wearable use among females, particularly for PA and mental health, might also be viewed through the HBM lens. Women’s greater focus on prevention could reflect a higher perceived susceptibility and severity of health risks, as well as stronger cues to action, such as societal expectations and health information targeting females [38]. In line with this, a previous study highlighted that females were more likely to use well-being apps to improve their health, whereas males were more interested in tracking their variables and tended to lose interest in these apps more quickly [39]. Moreover, factors influencing young adults’ adoption and use of apps for health purposes are shaped by socioeconomic status and educational level, creating discrepancies and barriers for individuals with low socioeconomic status and lower educational levels, even when they are interested in using them [40].

In contrast with other studies [30,41,42], a very high frequency of use was found among the majority of our sample (148/232, 63.8%, users of wearables for PA), who reported using mobile apps and wearables to measure their health variables on an everyday basis. According to the HBM, this preference may be due to the immediate perceived benefits of short-term feedback, which reinforces behavior change. However, 38 out of 144 (26.4%) participants reported the need for multiple apps to track PA, highlighting a potential barrier: fragmented tools may lower perceived practicality and increase user burden. Integrating comprehensive solutions that address all necessary variables could enhance adoption and self-efficacy. Moreover, it can be argued that the wider adoption of apps for PA might be due to their greater market presence, supported by the widespread promotion of well-known brands such as Nike or Puma. The daily use of apps and wearables may indicate their potential in health interventions that require frequent monitoring over time, as well as the possibility of tracking small daily results to keep users motivated toward long-term goals.

Survey respondents demonstrated a clear preference for wearables over apps, especially for PA and mental health tracking. According to HBM’s self-efficacy principle, the automatic data collection provided by wearables reduces the effort required for health monitoring. This ease of use increases confidence and adherence, as wearables simplify behavior tracking [43] while offering precise data [44]. Nonetheless, further analysis of the response ratios revealed a positive interest in the option of manually uploading data, which could represent an additional personalization feature of apps or wearables. Conversely, diet monitoring remains underutilized due to barriers such as manual input requirements, which reduce both perceived benefits and self-efficacy. Additionally, in our sample, wearables were also used to monitor sleep, mindfulness, and meditation practices. The latter is an interesting aspect, as another study [45] reported the use of a wearable to assess meditation practices, but only to identify abrupt movements during meditation rather than to monitor variables such as breathing or heartbeat. By contrast, participants in our sample reported using wearables to monitor actual meditation and mindfulness practices.

### Comparison With Prior Studies

Regarding the motivations for use in our population, apps and wearables were mostly adopted for monitoring health variables and, additionally, for receiving personal advice based on individual data collected. These results align with previous literature [31] on young populations [43], which also reported a similar rationale behind the use of technological tools to support health. Considering these motivations and the HBM theory, the importance of cues to action and personal advice emerges. Hence, based on the results obtained, health interventions for young adults might benefit from the use of apps and wearables to monitor health variables while also providing personalized advice to support daily tasks aimed at improving health outcomes over time, particularly in the PA domain. A study conducted on university students showed that PA apps, in particular, are valued and considered useful; therefore, people are more likely not only to use them but also to promote their use [46], further supporting the hypothesis that apps may be beneficial in long-term health interventions beginning in youth.

Characteristics of mobile apps and wearables identified as important by the respondents were (1) user-friendliness, (2) free access to all content, and (3) fast loading speed of the app/wearable and its contents. These elements are key enablers of technology adoption and should therefore be carefully addressed by app and wearable developers [47]. User-friendliness, in particular, may increase self-efficacy, tool effectiveness [48], and young people’s adoption of technology [49]. Previous evidence has already highlighted the importance of user-friendliness for young populations, especially in relation to fitness apps [50]. Moreover, free content may reduce financial barriers to access for people with low socioeconomic positions, thereby enabling the reach of broader populations [51,52], especially considering that app prices can vary depending on the specific app and market [53]. In line with this assumption, almost all participants were not interested in purchasing premium content. Interestingly, all the abovementioned aspects relate only to device characteristics and do not consider the specific types of content provided (ie, educational, motivational, informative) [38]. Regarding loading speed, this feature has been emphasized in previous literature [47,54,55] as a crucial element for technology acceptance, as users typically spend very little time deciding whether to use an app on their devices [56]. Hence, to ensure young adults’ use of apps and wearables in health interventions, the choice should favor fast-loading tools that are inexpensive or free, to minimize reported barriers.

Interestingly, only half of the sample acknowledged the importance of including educational content within apps [57,58], as well as the importance of technology assisting users in understanding the benefits of improving health variables [47]. Similar results were observed for the motivational aspect: half of the users considered it important that apps or wearables support them in improving their health. This may be considered controversial, given the nature of the apps and wearables investigated and previous studies reporting a positive attitude toward the technology’s supportive role in improving health variables [40,59]. Nonetheless, this finding is consistent with responses on motivations for use, which highlighted that the primary function of apps and wearables was perceived as monitoring health variables. While the HBM emphasizes that understanding the benefits of healthy behaviors can motivate action, these results indicate a need to better communicate such benefits through engaging, user-centered app features.

Regarding the aesthetics of apps and wearable devices, the overall design and graphical aspects were not considered as significant as the clarity of the icons [52,53]. This particular element has been highlighted as a crucial factor in attracting users to the technology [60]. Additionally, the possibility of personalizing the app or wearable interface was deemed quite important, in line with existing evidence [61-63]. Such personalization may enhance users’ perceived self-efficacy and reduce barriers to adoption, making the technology more adaptable to individual needs.

Despite evidence supporting gamification strategies for enhancing engagement and health outcomes [64], only one-third of our respondents expressed interest in playful features. This may suggest that young adults already feel sufficiently motivated to use these tools, reducing the perceived need for additional cues to action. A deeper analysis of the response ratios highlighted the significance of community functions. Previous literature has reported these features as a positive strategy for promoting challenging behaviors and motivation [65], serving as optional cues to action in health interventions. However, while participants in our study acknowledged community functions as important, they also reported not using them, raising questions about their actual role in motivating users to achieve their goals. In the literature, community features are often described as useful and appreciated in motivating individuals to improve their behaviors, particularly among young women [39], but mostly when they can view and compare results with people they personally know [50,66]. Nonetheless, a scoping review reported mixed results regarding their effectiveness in improving PA outcomes, largely due to the poor methodological quality of the studies evaluated [67]. Therefore, despite some positive results, further research is needed to strengthen the evidence on the use of community and social features in health programs targeting PA [68]. These features could be incorporated into health apps and wearables as optional tools, allowing users to decide whether to engage with them based on individual preferences. Thus, considering both the results obtained from our study and those of previous research, it would be useful for future studies to further investigate the effectiveness of community and social functions in young adult populations through both qualitative and quantitative approaches.

Given that mobile apps and wearables are frequently used by young adults for monitoring purposes, they could be integrated as valuable tools in strategies for early active aging [69,70]. These technologies may support the monitoring of health variables while also educating individuals about the importance of improving them. To further promote adoption and more efficient use of apps and wearables for health purposes, it is important to involve young adults in their design and development processes [63,71,72]. Finally, clear guidelines should be provided on how to develop effective health-related apps and wearables [73].

### Strengths and Limitations

A strength of this study is the specific population investigated and the broad inclusion criteria adopted, which allowed for a more accurate description of the patterns of young adults who use apps and wearables for health purposes. Moreover, the broad inclusion criteria enabled heterogeneity in participants’ characteristics, supporting the generalization of findings to the broader population. Another strength was the large number of questionnaires collected, which ensured sufficient statistical power to perform different types of analysis on the data and even examine subgroups (eg, gender). Despite this being the first study conducted in Italy on this population, some limitations should be acknowledged. First, the public representative who participated in the study was a student with prior professional experience in different roles (eg, social media manager, press officer). While her status as a student may have been an influencing factor, her communication skills and educational background provided a privileged perspective, making her contribution appropriate and constructive rather than biased. However, it cannot be excluded that her high level of education may limit the representativeness of individuals with lower educational backgrounds. Second, although this study investigated commercial mobile apps, the apps mentioned by participants were predominantly from renowned tech brands, with only a limited number of independent, nonprofit-oriented apps being reported. Future studies should aim to include a larger number of users of apps and wearables not linked to major brands to capture a broader range of opinions on different types of health tools. Third, data collection was conducted exclusively through digital procedures, which may have posed a barrier for individuals with poor digital literacy. However, given the young age of participants and the nature of the topic under investigation, such barriers were considered unlikely to represent a significant hindrance. Fourth, it cannot be excluded that, due to the digital nature of data collection, our sample may have been skewed toward individuals more comfortable with technology. Fifth, it is also possible that participants who completed the questionnaire were more interested in the topic investigated, which could have introduced distortion in the results. Sixth, the final sample included a high proportion of students, which may have influenced the findings; including more young adults who are not students might yield different results. Seventh, despite researchers’ efforts to ensure the reliability of responses (as explained in the “Methods” section), the anonymous nature of the web survey could have introduced inaccuracies and self-reporting bias. In particular, participants may have overestimated their healthy behaviors (eg, social desirability bias). However, given the large number of responses and the nature of the topic, it is unlikely that responses were falsified. This likelihood is further supported by the study’s aim, which focused on investigating the prevalence of app and wearable use rather than assessing their effectiveness. Eight, as it fell outside the study’s aims, objective health outcomes were not directly assessed in relation to app or wearable usage; instead, only the outcomes reported and collected by apps and wearables were considered, given that this was an exploratory study aimed at assessing the prevalence of use in young adults. Finally, socioeconomic status and geographic location data were not collected, making it impossible to examine their potential interactions with other variables. These aspects should be addressed in future research.

### Future Directions

Further studies should assess the motivation, expectations, and user experience associated with apps and wearables for health variables, exploring other aspects that young adults might find helpful in improving their health. Barriers and facilitators to the use of these technologies in young adults should also be investigated to strengthen the evidence base and enhance their adoption. Moreover, future research should involve a larger proportion of individuals not engaged in educational programs and include those with weaker digital skills. Similar prevalence assessments should also be conducted in other age groups, such as adults and older adults. Finally, studies targeting health in young adults, particularly improvements in PA, should prioritize wearables over apps.

### Conclusions

This study highlighted that young Italian adults track health-related variables more often via wearables than through apps, although both are consulted daily. Based on the results, young adults, particularly females, predominantly monitor PA-related variables, as these can be more easily tracked, while mental health and diet are monitored less. Moreover, apps and wearables are mainly adopted for monitoring rather than for improving health variables. Regarding the most important characteristics and features of these tools for young adults, user-friendliness, free access to all content, and the loading speed of the app/wearable software and content emerged as key. These characteristics should therefore be considered when developing or optimizing health interventions supported by apps and wearables in this population.

## Supplementary material

10.2196/64629 Multimedia Appendix 1 Survey.

10.2196/64629 Multimedia Appendix 2 Study advertisement.

10.2196/64629 Multimedia Appendix 3 Number of parameters monitored for physical activity, diet, and mental health; motivations for use of apps and wearables for physical activity, diet, and mental health.

10.2196/64629 Multimedia Appendix 4 Use of premium contents and community functions.

10.2196/64629 Multimedia Appendix 5 Binomial logistic regression results.

## Abbreviations

HBM: health belief model
mHealth: mobile health
STROBE: Strengthening the Reporting of Observational Studies in Epidemiology
