# Supplementary material for: Smartphone Apps and Wearables for Health Parameters in Young Adulthood: Cross-Sectional Study
Source: JMIR Hum Factors. 2025 Sep 3;12:e64629. doi: 10.2196/64629 (PMC12407497; doi:10.2196/64629)
Supplement: Multimedia Appendix 1 [file humanfactors-v12-e64629-s001.docx]

Supplementary Material 1

*Translated version of the questionnaire*

1. Section 1 – Demographic
2. Do I have between 18 and 26 years old included?
   1. Yes (go on with question 2)
   2. No (exit from the survey)
3. Age (insert number)
4. What gender do you identify with?
   1. Male
   2. Female
   3. Other (specify)
5. What is your height? (insert the answer in cm, ex. 180)
6. What is your weight? (insert the answer in kg, ex. 70)
7. Do you study at the University of Genoa? (PhD students are included as well)
   1. Yes
   2. No (go to question 9)
8. I am attending a:
   1. Bachelor of Science
   2. Master of Science
   3. PhD
9. The degree course you attend falls into the category:

a. Humanities and religious disciplines (history, philosophy...)

b. Applied and medical sciences (biotechnology, electronics, engineering, computer science, motor science...)

c. Mathematical, physical and natural sciences (astronomy, chemistry, biology...)

d. Humanities and social sciences (law, pedagogy, political science, economics...)

1. What is the last educational degree that you achieved?
   1. Primary high school diploma
   2. Secondary high school diploma
   3. Bachelor of Science
   4. Master of Science
   5. PhD
2. Section 2 – App and device/wearable for physical activity

App: smartphone applications

Device/wearable: wearable devices (ex. Smartwatch, smartband, fitness tracker)

1. Do you use at least one app or device/wearable to monitor physical activity?
   1. Yes
   2. No (go to Section 3)
2. Indicate which apps you use (you can select multiple options):
   1. WeWard
   2. Google Fit
   3. Adidas Running
   4. Samsung Health
   5. Komoot
   6. SweatCoin
   7. Zepp Life
   8. BuddyFit
   9. Other (insert answer)
   10. I only use the device/wearable (go to question 6)
3. Indicate which parameters you monitor with the apps: (you can select multiple options)
   1. Steps count
   2. Distance
   3. Weight
   4. Blood oxygen level/Oxygen saturation
   5. Daily activity
   6. Trainings
   7. Heart rate
   8. Body composition
   9. Other (insert answer)
4. I use the app for:
   1. Following a training plan
   2. Looking for and/or following walking/running/cycling paths
   3. Other (insert answer)
5. How often do you use the app?
   1. Everyday
   2. 5 – 6 times a week
   3. 3 - 4 times a week
   4. 1 o 2 times a week
   5. Once every 2 weeks
   6. Once a month
   7. Other (insert answer)
6. Indicate which device/wearable you use: (you can select multiple options)
   1. Apple Watch series 8
   2. Galaxy Watch 5 pro
   3. MiBand 6
   4. OPPO Watch
   5. TicWatch Pro 3
   6. Amazfit GTS 4
   7. Amazfit GTR 3
   8. Huawei Watch GT2
   9. Fossil ibrido HR
   10. Garmin Vivoactive 4
   11. Fitbit Versa 4
   12. Other (insert answer)
   13. I only use the app (go to Section 3)
7. Indicate which parameters you monitor with the device/wearable (you can select multiple options):
   1. Steps count
   2. Distance
   3. Weight
   4. Blood oxygen level/Oxygen saturation
   5. Daily activity
   6. Trainings
   7. Heart rate
   8. Body composition
   9. Other (insert answer)
8. I use the device/wearable for:
9. Following a training plan
10. Looking for and/or following walking/running/cycling paths
11. Other (insert answer)
12. How often do you use the device/wearable?
    1. Everyday
    2. 5 – 6 times a week
    3. 3 - 4 times a week
    4. 1 o 2 times a week
    5. Once every 2 weeks
    6. Once a month
    7. Other (insert answer)
13. Do you use paid features on the app or device/wearable?
    1. Yes (insert which)
    2. No (insert why)
14. Do you use community functions?
    1. Yes
    2. No
15. Section 3 – App and device/wearable for diet

App: smartphone applications

Device/wearable: wearable devices (ex. Smartwatch, smartband, fitness tracker)

1. Do you use at least one app or device/wearable to monitor diet?
   1. Yes
   2. No (go to Section 4)
2. Indicate which apps you use (you can select multiple options):
   1. MyFitnesspal
   2. Fatsecret
   3. Yazio
   4. Melarossa
   5. Google Fit
   6. Food Advisor
   7. Samsung Health
   8. Calorie counter by Lose it!
   9. Other (insert answer)
   10. I only use the device/wearable (go to question 6)
3. Indicate which parameters you monitor with the apps (you can select multiple options):
   1. Calorie intake
   2. Water
   3. Weight
   4. Body composition
   5. Other (insert answer)
4. I use the app for:
   1. Following a meal plan
   2. Other (insert answer)
5. How often do you use the app?
   1. Everyday
   2. 5 – 6 times a week
   3. 3 - 4 times a week
   4. 1 o 2 times a week
   5. Once every 2 weeks
   6. Once a month
   7. Other (insert answer)
6. Indicate which device/wearable you use (you can select multiple options):
   1. Apple Watch series 8
   2. Galaxy Watch 5 pro
   3. MiBand 6
   4. OPPO Watch
   5. TicWatch Pro 3
   6. Amazfit GTS 4
   7. Amazfit GTR 3
   8. Huawei Watch GT2
   9. Fossil hybrid HR
   10. Garmin Vivoactive 4
   11. Fitbit Versa 4
   12. Other (insert answer)
   13. I only use the app (go to Section 4)
7. Indicate which parameters you monitor with the device/wearable (you can select multiple options):
   1. Calorie intake
   2. Water
   3. Weight
   4. Body composition
   5. Other (insert answer)
8. I use the device/wearable for:
   1. Following a meal plan
   2. Other (insert answer)
9. How often do you use the device/wearable?
10. Everyday
11. 5 – 6 times a week
12. 3 - 4 times a week
13. 1 o 2 times a week
14. Once every 2 weeks
15. Once a month
16. Other (insert answer)

10) Do you use paid features on the app or device/wearable?

1. Yes (insert which)
2. No (insert why)

11) Do you use community functions?

1. Yes
2. No
3. Section 4 – App and device/wearable for mental health

App: smartphone applications

Device/wearable: wearable devices (ex. Smartwatch, smartband, fitness tracker)

1. Do you use at least one app or device/wearable to monitor mental health?
   1. Yes
   2. No (go to Section 5)
2. Indicate which apps you use (you can select multiple options):
   1. Calm
   2. Headspace
   3. Serenity
   4. MindShift
   5. Samsung Health
   6. Breathe2Relax
   7. Self-help Anxiety Management (SAM)
   8. Other (insert answer)
   9. I only use the device/wearable (go to question 6)
3. Indicate which parameters you monitor with the app (you can select multiple options):
   1. Stress
   2. Sleep
   3. Meditation
   4. Breathing
   5. Other (insert answer)
4. I use the app for:
   1. Performing exercises of relaxation/meditation
   2. Following advice
   3. Other (insert answer)
5. How often do you use the app?
   1. Every day
   2. 5 – 6 times a week
   3. 3 - 4 times a week
   4. 1 o 2 times a week
   5. Once every 2 weeks
   6. Once a month
   7. Other (insert answer)
6. Indicate which device/wearable you use (you can select multiple options):
   1. Apple Watch series 8
   2. Galaxy Watch 5 pro
   3. MiBand 6
   4. OPPO Watch
   5. TicWatch Pro 3
   6. Amazfit GTS 4
   7. Amazfit GTR 3
   8. Huawei Watch GT2
   9. Fossil hybrid HR
   10. Garmin Vivoactive 4
   11. Fitbit Versa 4
   12. Other (insert answer)
   13. I only use the app (go to Section 5)
7. Indicate which parameters you monitor with the device/wearable (you can select multiple options):
   1. Stress
   2. Sleep
   3. Meditation
   4. Breathing
   5. Other (insert answer)
8. I use the device/wearable for:
   1. Performing exercises of relaxation/meditation
   2. Following advice
   3. Other (insert answer)
9. How often do you use the device/wearable?
10. Everyday
11. 5 – 6 times a week
12. 3 - 4 times a week
13. 1 o 2 times a week
14. Once every 2 weeks
15. Once a month
16. Other (insert answer)

10) Do you use paid features on the app or device/wearable?

1. Yes (insert which)
2. No (insert why)

11) Do you use community functions?

1. Yes
2. No
3. Section 5 – Preferences in apps and devices/wearables

How much is important for you:

| Question | Not important at all | Little important | Neutral | Quite important | Extremely important |
| --- | --- | --- | --- | --- | --- |
| 1. The fact that the app or device/wearable helps me effectively manage my health and well-being |  |  |  |  |  |
| 1. The fact that the app/device motivates me to change my lifestyle in a positive way |  |  |  |  |  |
| 1. The fact that the app/device helps me to improve my lifestyle without letting me fully understand the benefits |  |  |  |  |  |
| 1. Presence of educational content within the app/device |  |  |  |  |  |
| 1. The design/the graphics |  |  |  |  |  |
| 1. Icon clarity |  |  |  |  |  |
| 1. The possibility to customize the app/device from a graphic point of view |  |  |  |  |  |
| 1. The ease of use |  |  |  |  |  |
| 1. The loading speed |  |  |  |  |  |
| 1. The possibility to download data from the app/device in formats such as pdf or excel |  |  |  |  |  |
| 1. The possibility to manually upload data to the app/device |  |  |  |  |  |
| 1. Not having community functions |  |  |  |  |  |
| 1. Get all the app/device contents for free |  |  |  |  |  |
| 1. Get the chance to buy premium content that others don’t have |  |  |  |  |  |
| 1. The possibility to have a playful approach with the app (ex. forest o plant nanny) |  |  |  |  |  |
