# Supplementary figures and images for: Smartphone Apps and Wearables for Health Parameters in Young Adulthood: Cross-Sectional Study
Source: JMIR Hum Factors. 2025 Sep 3;12:e64629. doi: 10.2196/64629 (PMC12407497; doi:10.2196/64629)

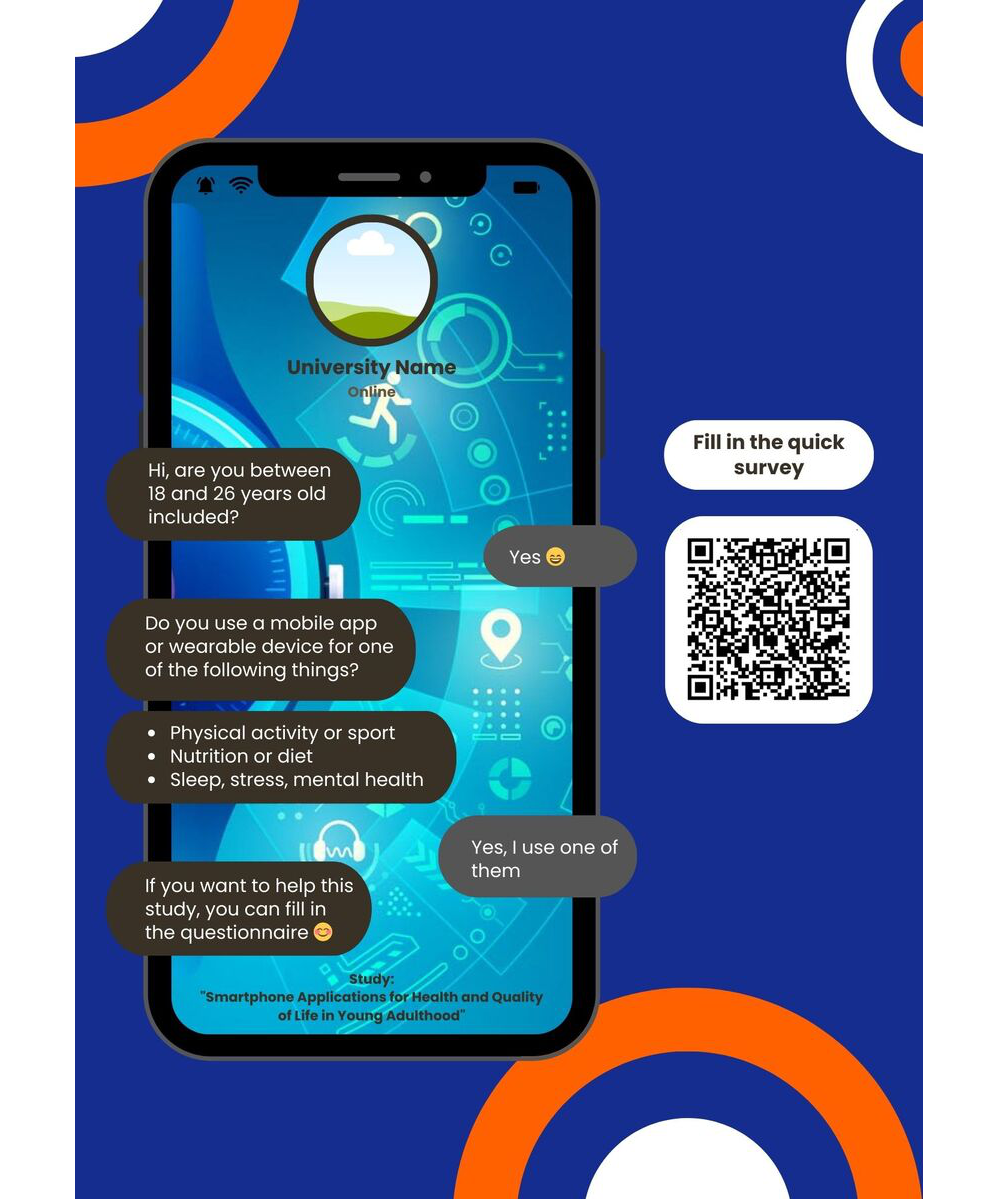

Supplement: Multimedia Appendix 2 [file humanfactors-v12-e64629-s002.png]

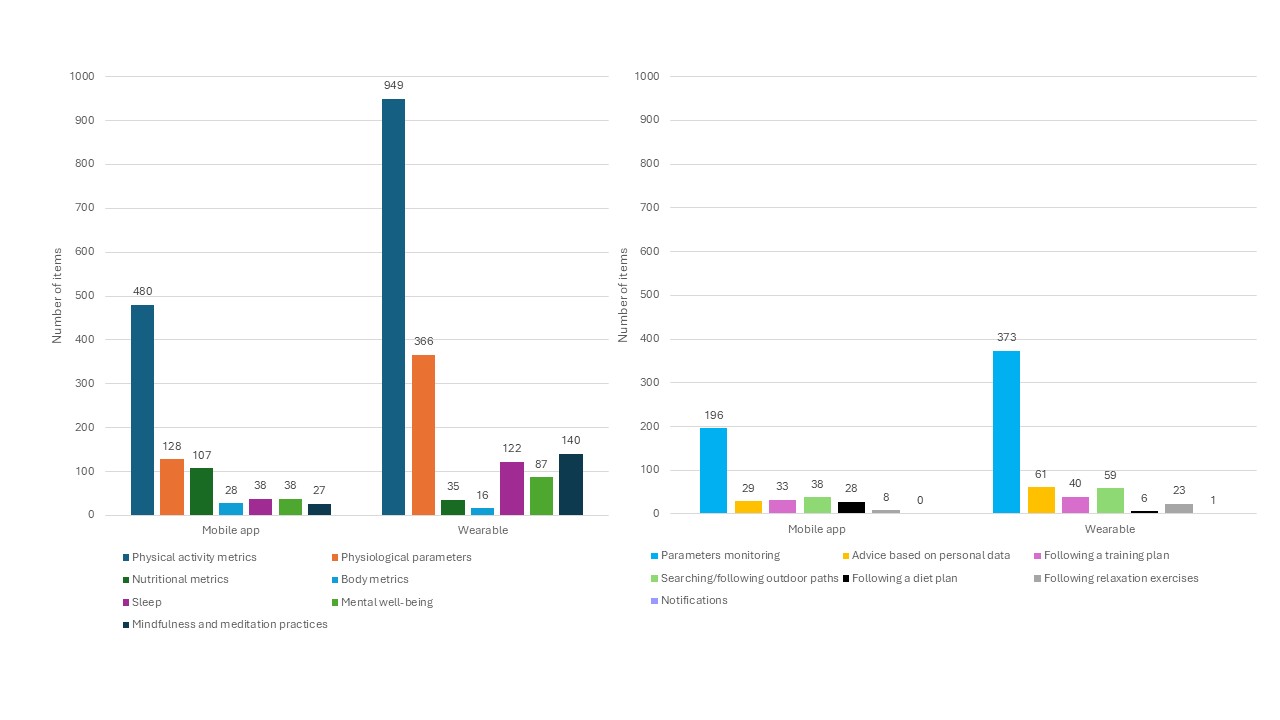

Supplement: Multimedia Appendix 3 [file humanfactors-v12-e64629-s003.jpg]
