# Supplementary material for: Smartphone Apps and Wearables for Health Parameters in Young Adulthood: Cross-Sectional Study
Source: JMIR Hum Factors. 2025 Sep 3;12:e64629. doi: 10.2196/64629 (PMC12407497; doi:10.2196/64629)
Supplement: Multimedia Appendix 4 [file humanfactors-v12-e64629-s004.docx]

Supplementary Material 2

*Table 6 – Use of premium contents and community functions*

|  | Physical Activity (N = 320) | | Diet (N = 60) | | Mental Health (N = 156) | |
| --- | --- | --- | --- | --- | --- | --- |
|  | Yes | No | Yes | No | Yes | No |
| Premium contents | 10 (3,1%) | 310 (96,9%) | 2 (3,3%) | 58 (96,7%) | 4 (2,6%) | 152 (97,4%) |
| Community functions | 31 (8,7%) | 289 (90,3%) | 6 (10%) | 54 (90%) | 14 (9%) | 142 (91%) |

*Legend: PA, physical activity; MH, mental health.*
