# Supplementary material for: Smartphone Apps and Wearables for Health Parameters in Young Adulthood: Cross-Sectional Study
Source: JMIR Hum Factors. 2025 Sep 3;12:e64629. doi: 10.2196/64629 (PMC12407497; doi:10.2196/64629)
Supplement: Multimedia Appendix 5 [file humanfactors-v12-e64629-s005.docx]

**Supplementary material**

# Binomial logistic regressions for apps’ use based on gender

Coefficient of the Model – Use of at least one app for Physical Activity

**95% Confidence Interval**

**Predictor Estimate SE t p Odds-Ratio Min Max**

| Intercept | -0.385 | 0.107 | -3.5993 | < .001 | 0.681 | 0.552 | 0.839 |
| --- | --- | --- | --- | --- | --- | --- | --- |
| Gender: |  |  |  |  |  |  |  |
| Male – Female | 0.710 | 0.155 | 4.5904 | < .001 | 2.034 | 1.502 | 2.754 |
| Non binary – Female | -13.181 | 535.411 | -0.0246 | 0.980 | 1.89e-6 | 0.000 | Inf |

*Legend: SE, standard error; R^2^=0.024*

| Coefficient of the Model – Use of at least one app for Diet | | | | | | | | | | | | | | | |
| --- | --- | --- | --- | --- | --- | --- | --- | --- | --- | --- | --- | --- | --- | --- | --- |
|  | | | | | | | | | | | | **95% Confidence Interval** | | | |
| **Predictor** | | **Estimate** | | **Standard Error** | | **t** | | **p** | | **Odds-Ratio** | | **Min** | | **Max** | |
| Intercept |  | 2.1462 |  | 0.171 |  | 12.5187 |  | < .001 |  | 8.55 |  | 6.112 |  | 11.97 |  |
| Gender: |  |  |  |  |  |  |  |  |  |  |  |  |  |  |  |
| Male – Female |  | 0.0818 |  | 0.253 |  | 0.3231 |  | 0.747 |  | 1.09 |  | 0.661 |  | 1.78 |  |
| Non binary – Female |  | -15.7123 |  | 535.411 |  | -0.0293 |  | 0.977 |  | 1.50e-7 |  | 0.000 |  | Inf |  |
| *Legend: R^2^=0.010* | | | | | | | | | | | | | | | |
|  | | | | | | | | | | | | | | | |

| Coefficient of the Model – Use of at least one app for Mental Health | | | | | | | | | | | | | | | |
| --- | --- | --- | --- | --- | --- | --- | --- | --- | --- | --- | --- | --- | --- | --- | --- |
|  | | | | | | | | | | | | **95% Confidence Interval** | | | |
| **Predittore** | | **Estimate** | | **Standard Error** | | **t** | | **p** | | **Odds-Ratio** | | **Min** | | **Max** | |
| Intercept |  | 0.899 |  | 0.116 |  | 7.7662 |  | < .001 |  | 2.46 |  | 1.96 |  | 3.08 |  |
| Gender: |  |  |  |  |  |  |  |  |  |  |  |  |  |  |  |
| Male – Female |  | 0.522 |  | 0.181 |  | 2.8815 |  | 0.004 |  | 1.69 |  | 1.18 |  | 2.40 |  |
| Non binary – Female |  | 12.667 |  | 535.411 |  | 0.0237 |  | 0.981 |  | 317130.06 |  | 0.00 |  | Min |  |
| *Legend: R^2^=0.011* | | | | | | | | | | | | | | | |
|  | | | | | | | | | | | | | | | |

# Binomial logistic regression for apps’ use based on level of education

| Coefficient of the Model – Use of at least one app for Physical Activity | | | | | | | | | | | | | | | |
| --- | --- | --- | --- | --- | --- | --- | --- | --- | --- | --- | --- | --- | --- | --- | --- |
|  | | | | | | | | | | | | **95% Confidence Interval** | | | |
| **Predictor** | | **Estimate** | | **Standard Error** | | **t** | | **p** | | **Odds-Ratio** | | **Min** | | **Max** | |
| Intercept |  | 0.0870 |  | 0.0984 |  | 0.884 |  | 0.376 |  | 1.091 |  | 0.900 |  | 1.323 |  |
| Educational level: |  |  |  |  |  |  |  |  |  |  |  |  |  |  |  |
| Master degree – High school diploma |  | -0.5978 |  | 0.5257 |  | -1.137 |  | 0.255 |  | 0.550 |  | 0.196 |  | 1.541 |  |
| Bachelor degree – High school diploma |  | -0.9743 |  | 0.1962 |  | -4.966 |  | < .001 |  | 0.377 |  | 0.257 |  | 0.554 |  |
| Secondary school diploma – High school diploma |  | 0.7855 |  | 0.2456 |  | 3.198 |  | 0.001 |  | 2.193 |  | 1.355 |  | 3.550 |  |
| *Legend: R^2^=0.050* | | | | | | | | | | | | | | | |
|  | | | | | | | | | | | | | | | |

| Coefficient of the Model – Use of at least one app for Diet | | | | | | | | | | | | | | | |
| --- | --- | --- | --- | --- | --- | --- | --- | --- | --- | --- | --- | --- | --- | --- | --- |
|  | | | | | | | | | | | | **95% Confidence Interval** | | | |
| **Predictor** | | **Estimate** | | **Standard Error** | | **t** | | **p** | | **Odds-Ratio** | | **Min** | | **Max** | |
| Intercept |  | 2.1041 |  | 0.158 |  | 13.3258 |  | < .001 |  | 8.200 |  | 6.017 |  | 11.17 |  |
| Educational level: |  |  |  |  |  |  |  |  |  |  |  |  |  |  |  |
| Master degree – High school diploma |  | -0.1582 |  | 0.772 |  | -0.2049 |  | 0.838 |  | 0.854 |  | 0.188 |  | 3.88 |  |
| Bachelor degree – High school diploma |  | 0.0161 |  | 0.295 |  | 0.0546 |  | 0.956 |  | 1.016 |  | 0.570 |  | 1.81 |  |
| Secondary school diploma – High school diploma |  | 0.5927 |  | 0.450 |  | 1.3161 |  | 0.188 |  | 1.809 |  | 0.748 |  | 4.37 |  |
| *Legend: R^2^=0.005* | | | | | | | | | | | | | | | |
|  | | | | | | | | | | | | | | | |

| Coefficient of the Model – Use of at least one app for Mental Health | | | | | | | | | | | | | | | |
| --- | --- | --- | --- | --- | --- | --- | --- | --- | --- | --- | --- | --- | --- | --- | --- |
|  | | | | | | | | | | | | **95% Confidence Interval** | | | |
| **Predictor** | | **Estimate** | | **Standard Error** | | **t** | | **p** | | **Odds-Ratio** | | **Min** | | **Max** | |
| Intercept |  | 1.198 |  | 0.116 |  | 10.285 |  | < .001 |  | 3.312 |  | 2.637 |  | 4.162 |  |
| Educational level: |  |  |  |  |  |  |  |  |  |  |  |  |  |  |  |
| Master degree – High school diploma |  | -0.409 |  | 0.552 |  | -0.742 |  | 0.458 |  | 0.664 |  | 0.225 |  | 1.959 |  |
| Bachelor degree – High school diploma |  | -0.531 |  | 0.200 |  | -2.652 |  | 0.008 |  | 0.588 |  | 0.397 |  | 0.871 |  |
| Secondary school diploma – High school diploma |  | 0.835 |  | 0.341 |  | 2.448 |  | 0.014 |  | 2.305 |  | 1.181 |  | 4.499 |  |
| *Legend: R^2^=0.024* | | | | | | | | | | | | | | | |
|  | | | | | | | | | | | | | | | |
